# Stent-Retriever Thrombectomy in STEMI With Large Thrombus Burden: The RETRIEVE AMI Randomized Trial

**DOI:** 10.1016/j.jacadv.2025.101893

**Published:** 2025-06-23

**Authors:** Rafail A. Kotronias, Jason L. Walsh, Stefano Andreaggi, Leonardo Portolan, Alessandro Maino, Federico Marin, Jason Chai, Ikboljon Sobirov, Muhammad Sheikh, Thomas J. Cahill, Andrew J. Lucking, Max Costello, Eva Fraile Moreno, Vrinda Haridas, Anisha Shaji, Hector M. Garcia-Garcia, Keith M. Channon, Adrian P. Banning, Jeremy P. Langrish, Giovanni Luigi De Maria, OxAMI and RETRIEVE AMI Investigators

**Affiliations:** a Oxford University Hospitals NHS Foundation Trust, John Radcliffe Hospital, Oxford, United Kingdom; b Acute Multidisciplinary Imaging & Interventional Centre (AMIIC), Division of Cardiovascular Medicine, Radcliffe Department of Medicine, University of Oxford, Oxford, United Kingdom; c Division of Interventional Cardiology, MedStar Cardiovascular Research Network, MedStar Washington Hospital Center, Washington, DC, USA

**Keywords:** large thrombus burden, optical coherence tomography (OCT), STEMI, stent-retriever thrombectomy, thrombus modification

## Abstract

**Background:**

Percutaneous coronary intervention (PCI) restores epicardial flow in ST-segment elevation myocardial infarction (STEMI), but large thrombus burden (LTB) can impair myocardial perfusion due to embolization. While manual aspiration (MA) devices have limited efficacy in STEMI, the success of stent-retriever thrombectomy (SRT) in stroke suggests it as a promising option for STEMI.

**Objectives:**

The RETRIEVE AMI (stent-retriever thrombectomy for thrombus burden reduction in patients with acute myocardial infarction) trial assessed the safety and efficacy of Solitaire X SRT vs Export MA in STEMI patients with LTB.

**Methods:**

This single-center study enrolled 81 STEMI patients with LTB (TIMI thrombus grade ≥4) and randomized them to PCI, MA-assisted, or SRT-assisted PCI. The primary endpoint was difference in prestent thrombus volume by optical coherence tomography between SRT and either comparator; coprimary endpoints included device-related target vessel complications and major adverse cardiac and cerebrovascular events through 6 months.

**Results:**

SRT was performed in 26 cases (one crossover), and MA in 27. No device-related arterial complications or cerebrovascular events occurred in the SRT arm. Baseline thrombus volume was significantly higher in the SRT group (18.3 mm^3^) compared to MA (7.7 mm^3^) and no modification (9.8 mm^3^; *P* = 0.04). Prestent thrombus volume was not significantly different between SRT (7.7; IQR: 2.3-18.6) and either MA (4.8; IQR: 1.8-8.4; *P* = 0.17) or no thrombus modification (9.8; IQR: 4.5-18.1; *P* = 1.00). Both techniques significantly reduced prestent thrombus burden (SRT: 12.8%; IQR: 4.4%-21.5%; *P* = 0.016 and MA: 13.0%; IQR: 3.8%-19.4%; *P* = 0.003) compared to no modification (22.8%; IQR: 10.4%-27.7%). No device-related clinically relevant arterial injury was detected and in-hospital and 6-month major adverse cardiac and cerebrovascular events did not differ between arms.

**Conclusions:**

RETRIEVE AMI demonstrates the feasibility of Solitaire X SRT in STEMI with LTB. Prestent thrombus volume was not different between SRT, MA, or no thrombus modification, although SRT extracted larger thrombus volume than MA. Larger multicenter studies using optical coherence tomography-based criteria are needed to minimize variability and enhance comparative assessments.

In ST-segment elevation myocardial infarction (STEMI), primary percutaneous coronary intervention (PCI) effectively restores epicardial coronary flow, yet suboptimal myocardial perfusion often persists post primary PCI resulting in prognostically relevant myocardial injury. Embolization of atherothrombotic material is an important contributor to this injury, particularly when the angiographic thrombus burden is large.^1^

Interventional techniques have been developed to modify (extract or dissipate) coronary thrombus in STEMI, aiming to reduce thrombus embolization, microvascular injury, and myocardial injury.^2^ Manual thrombus aspiration is the main thrombus modification technique used in current clinical practice. However, large randomized controlled trials found no improvement in angioplasty result^3^ or major adverse cardiac events with the routine use of manual aspiration (MA) during STEMI.^4^ Hence, its routine use during STEMI is not recommended.

The disappointing results of MA trials in STEMI may stem from inadequate patient selection, as these trials included all STEMI cases regardless of thrombus burden. A patient-level meta-analysis focusing on cases with a large thrombus burden (LTB) supports this, suggesting a nominal reduction in major adverse cardiovascular events with MA.^4^ Another likely factor is the advocated limited thrombus extraction efficacy of MA, as studies report a high failure rate and comparable coronary thrombus volumes between treated and untreated patients.^5^^,^^6^ The limited efficacy of manual thrombus aspiration, coupled with the poor prognosis associated with LTB in STEMI, highlights an unmet clinical need for more effective thrombus extraction strategies in this setting.^7^

A thrombus modification renaissance has occurred in stroke intervention, with stent-retriever thrombectomy (SRT) being shown to have clear clinical efficacy for the treatment of ischemic stroke in multiple randomized studies.^8^ Currently, only case reports and series demonstrate the feasibility of stent-retriever use in STEMI with LTB.^9^^,^^10^ It remains unclear if SRT can efficaciously remove thrombus in patients with STEMI.

In this randomized study, we evaluate the safety and thrombus extraction efficacy of SRT (Solitaire X revascularization device, Medtronic, Inc) compared to MA (Export, Medtronic, Inc) and assess the impact of thrombus modification on myocardial perfusion in STEMI patients with LTB.

## Methods

### Trial design and participants

**Figure 1:**
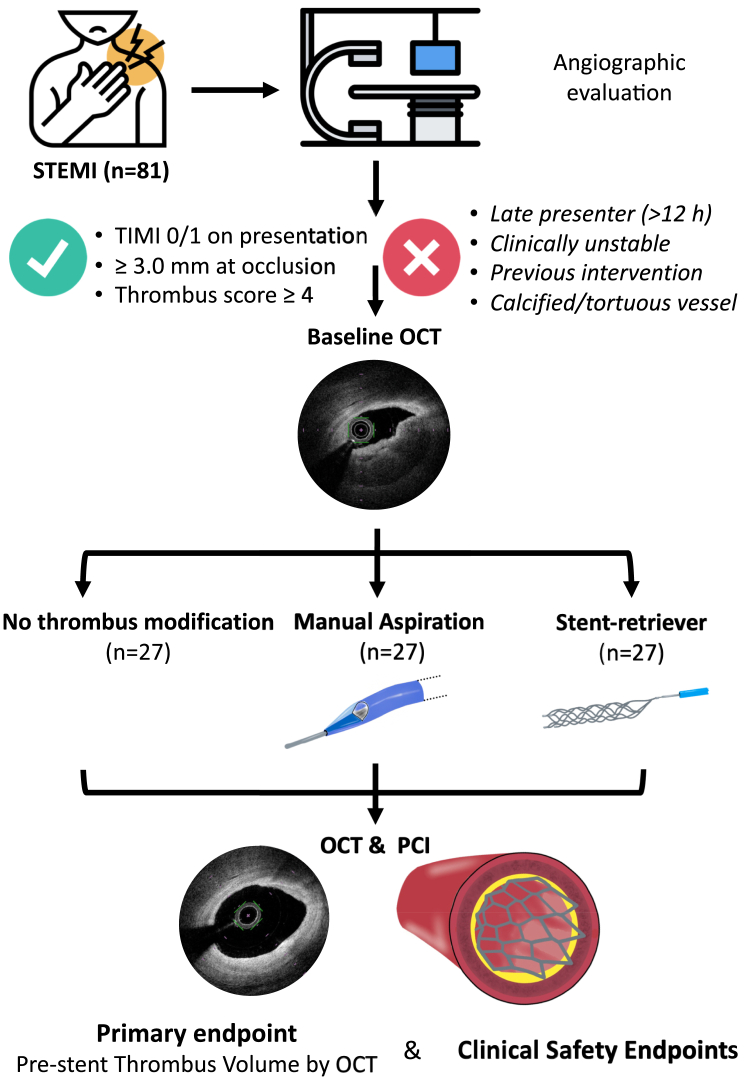
The RETRIEVE AMI Study Methodology OCT = optical coherence tomography; PCI = percutaneous coronary intervention; RETRIEVE AMI = stent-retriever thrombectomy for thrombus burden reduction in patients with acute myocardial infarction; STEMI = ST-segment elevation myocardial infarction.

The RETRIEVE AMI (stent-retriever thrombectomy for thrombus burden reduction in patients with acute myocardial infarction) is an investigator-initiated, prospective, single-center, randomized, controlled trial performed at the Oxford Heart Centre from May 6, 2022, to October 10, 2024. The RETRIEVE AMI study enrolled participants presenting with STEMI and angiographically LTB after wiring (TIMI thrombus grade ≥4) in any coronary artery with reference diameter ≥3 mm. Exclusion criteria are listed in the supplementary material. Of 213 screened patients, 81 participants were enrolled and randomized 1:1:1 to receive one of: 1) stand-alone PCI; 2) MA-assisted PCI; and 3) SRT-assisted PCI. The detailed participant flow diagram is presented in Supplemental Figure 1 and the study flow is illustrated in Figure 1.

The trial design and methods of RETRIEVE AMI (NCT05307965) have been previously published.^11^ The trial was approved by the South Central Oxford C Ethics Committee (21/SC/0220) and the Medicines and Healthcare products Regulatory Agency (CI/2021/0077/GB), sponsored by the host institution and conducted in accordance with the principles of the Declaration of Helsinki, Good Clinical Practice, and in full conformity with European Commission Medical Device Guidelines and Guide to European Medical Device Trials and BS EN ISO 14155. The study protocol was amended in 2023 to include patients with STEMI affecting the left coronary system, with 8 participants included by then. All participants were verbally assented and written informed consent was sought as soon as the participant’s clinical condition allowed (see Supplemental Appendix for further information).

### Randomization process

A random allocation sequence was generated with R Studio (*randomizeR* package) and concealed using sequentially numbered opaque sealed envelopes by an individual independent to the study. Following enrollment by the research team, participants were assigned to an arm by unsealing the next available envelope.

### Percutaneous coronary intervention

Prehospital dual antiplatelet loading with aspirin 300 mg and clopidogrel 600 mg was administered in all patients. PCI was performed according to institutional guidelines. In brief, 7Fr or 7.5 Fr sheathless guide catheters were used. Systemic heparinization with 100 IU/kg was commenced and monitored by activated clotting time. Flow was restored with workhorse wires and eligibility evaluated prior to assent and randomization. Predilation with a 1.5 mm semicompliant balloon (upsized to 2.0 mm if vessel recoil occurred) at low pressure was performed to enable intravascular imaging with optical coherence tomography (OCT).^12^ The procedure progressed according to the allocation. OCT-guided angioplasty was performed according to institutional guidelines and following established principles.^13^ Lesion preparation and adjunctive pharmacotherapy (following thrombus modification) were left to the operator’s discretion.

### No thrombus modification study arm

Following intravascular imaging with OCT, angioplasty was performed according to institutional guidelines.

### Manual aspiration thrombectomy study arm

Following intravascular imaging with OCT, thrombus MA was performed with a commercially available aspiration catheter (Export Catheter, Medtronic). Per-protocol, 2 attempts at thrombectomy were performed. Each attempt comprised 3 passes of the aspiration catheter through the coronary occlusion site. OCT imaging was repeated after thrombus modification and intravascular imaging-guided angioplasty was performed according to institutional guidelines.

### Stent-retriever thrombectomy study arm

SRT was performed according to the stent-retriever-assisted vacuum-locked extraction (SAVE) technique.^14^ Operators underwent training conducted by a specialist of the device manufacturer, supplemented with hands-on experience with the device and procedural technique using a bench model.^15^ Following intravascular imaging, a 6-F extension guide catheter (Guidezilla, Boston Scientific) was advanced beyond the ostium of the culprit artery and a second workhorse wire was advanced to the distal vessel to enable prompt angioplasty following mechanical thrombectomy. Then, an over-the-wire Phenom microcatheter (Medtronic) was advanced on the first guidewire and positioned distal to thrombotic occlusion. The Solitaire X Revascularization Device (Medtronic) was advanced through the microcatheter until the distal radio-opaque markers reached the radio-opaque tip of the microcatheter. Once a satisfactory position was confirmed, the microcatheter was retracted into the extension guide catheter while forward tension on the device push wire was maintained. The position of the device was confirmed on fluoroscopy and the device was left in situ for 3 minutes. The device was withdrawn into the extension guide catheter under sustained aspiration, achieved with 3 VacLok syringes.^14^ A maximum of 2 deployments of the device were allowed. OCT imaging was repeated after thrombus modification and intravascular imaging-guided angioplasty was performed afterward according to institutional guidelines.

### Endpoints

The primary endpoint was prestent thrombus volume (mm^3^) evaluated by OCT imaging. For the no thrombus modification arm, the baseline thrombus volume was considered the prestent measurement. The primary prespecified comparisons were: 1) SRT vs MA thrombectomy; and 2) SRT vs no thrombus modification. Prespecified coprimary endpoints included: 1) angiography/OCT-defined device-related target vessel complications (dissections or perforation); and 2) major adverse cardiac and cerebrovascular events (MACCE) through 6 months. Secondary endpoints were: 1) angiographic myocardial perfusion indices; and 2) post-PCI residual thromboatheroma volume. Sensitivity analyses exploring changes in thrombus volume and burden were also performed. Further information on definitions, adjudication, and ascertainment of device-related target vessel complications and MACCE is presented in Supplemental Appendix. All outcomes were adjudicated by investigators unaware of the allocation of participants, while MACCE were adjudicated by an independent clinical event committee.

### Optical coherence tomography acquisition & analysis

OCT imaging was performed using either the LUNAWAVE imaging catheter (Terumo Corporation) or the Dragonfly OpStar Imaging catheter (Abbott Medical). OCT was performed at baseline, post thrombus modification, and post stent deployment. Catheters were calibrated prior to acquisition. Contrast was manually injected, and pullbacks were automatically triggered. The images obtained were reviewed by the operator to ensure adequate imaging quality and repeated when required for up to 3 times. Frame by frame OCT analyses for lumen parameters, thrombus quantification, and post angioplasty optimization were performed offline with QIVUS software (Medis Medical Imaging Systems) by a core laboratory team comprising 3 readers with >2 years of experience (S.A., J.W., A.M.) following the methodology adopted in the TOTAL OCT substudy and described in detail in the Supplemental Material.^12^ Comparative analyses were performed in matched segments. All analyses were reviewed by 2 readers with >5 years of experience in OCT (R.A.K., G.D.M.). Equivocal cases were adjudicated by consensus. All readers were unaware of a participant’s allocation and the timing (baseline/post thrombus modification) of the analyzed run. Interobserver and intraobserver reliability were performed on a per-slice basis in 5 randomly selected cases.

### Myocardial perfusion evaluation

Myocardial perfusion was evaluated by validated angiography derived indices; TIMI flow, corrected TIMI frame count, myocardial blush grade (MBG), rate of no reflow (TIMI flow <3 or MBG <3), and the angiography derived index of microvascular resistance (IMR_angio_).^16^ Furthermore, 12-lead electrocardiograms (ECGs) obtained 60 to 90 minutes following the procedure were analyzed for completeness of sum ST-segment resolution (>70% considered complete). Analyses were performed by a core laboratory team comprising 3 readers with >5 years of experience (S.A., J.W., R.A.K.), blinded to the treatment allocation. Equivocal cases were adjudicated by consensus.

### Statistical methods

Based on previously published work,^5^^,^^17^ we anticipated a mean prestent thrombus volume of 9.6 mm^3^ with a SD of 5.1 mm^3^. In the absence of specific data for SRT, we hypothesized that prestent thrombus volume would be 33% less in the SRT arm. Using a 1-way pairwise analysis of variance with a 2-sided equality, accounting for 2 pairwise comparisons (SRT vs MA thrombectomy and SRT vs no thrombus modification) with an α of 0.05, a sample size of 72 participants would have 80% power to detect a 33% difference in prestent thrombus volume with SRT. The final sample size of 81 accounted for an anticipated 12.5% dropout due to nonanalyzable or unavailable OCT imaging.

Statistical analyses were conducted using SPSS 29 (IBM Corp) and R studio (*ggplot2*). Continuous data are expressed as mean ± SD or as median (IQR), and categorical variables are presented as percentages or total numbers. Correlations were explored graphically and using the Spearman correlation coefficient. The primary endpoint was evaluated by the Kruskal-Wallis test with Bonferroni correction for the 2 prespecified comparisons. Chi-square tests or Fisher exact tests were used for coprimary and categorical secondary endpoints. Continuous secondary endpoints were compared with the Kruskal-Wallis test with Bonferroni correction. Sensitivity analyses employed the Kruskal-Wallis test with Bonferroni correction when >2 groups were compared, the Mann-Whitney *U* test or Moses’ test when 2 groups were compared and the Wilcoxon rank sum test for within-group comparisons. Results were analyzed and reported on a per-protocol basis for the primary endpoint. Coprimary and secondary endpoints were analyzed and reported on an intention to treat basis. A 2-sided *P* value ≤0.05 was considered statistically significant.

## Results

### Participants

A total of 81 patients were recruited and randomized. Participant flow is detailed in Supplemental Figure 1. Baseline demographic and anatomical characteristics and ischemic time were well balanced across all arms and representative of contemporary STEMI patients (Table 1). Illustrative cases are presented in Central Illustration A and Supplemental Figure 2.

**Table 1:** Baseline Characteristics

	Overall (N = 81)	No Thrombus Modification (n = 27)	Manual Aspiration (n = 27)	Stent-Retriever Thrombectomy (n = 27)
Demographics				
Age, y	64 ± 12	67 ± 12	63 ± 12	63 ± 13
Female, n (%)	12 (15%)	3 (11%)	5 (19%)	4 (15%)
Hypertension, n (%)	35 (43%)	13 (48%)	11 (41%)	11 (41%)
Hyperlipidemia, n (%)	22 (27%)	7 (26%)	8 (30%)	7 (26%)
Previous MI, n (%)	3 (4%)	3 (11%)	0 (0%)	0 (0%)
Current smoker, n (%)	20 (25%)	5 (19%)	8 (30%)	7 (26%)
Ischemia time, min	200 (145-338)	217 (161-383)	200 (142-293)	180 (144-252)
Procedural				
Antiplatelet loading, n (%)	81 (100%)	27 (100%)	27 (100%)	27 (100%)
Culprit, n (%)				
LAD	31 (38%)	14 (52%)	7 (26%)	10 (37%)
LCx	9 (11%)	2 (7%)	5 (18%)	2 (7%)
RCA	41 (51%)	11 (41%)	15 (56%)	15 (56%)
TIMI flow, n (%)				
0	76 (94%)	25 (93%)	26 (96%)	25 (93%)
1	5 (6%)	2 (7%)	1 (4%)	2 (7%)
TIMI thrombus grade, n (%)				
4, n (%)	34 (42%)	11 (41%)	12 (44%)	11 (41%)
5, n (%)	47 (58%)	16 (59%)	15 (56%)	16 (59%)

LAD = left anterior descending; LCx = left circumflex; MI = myocardial infarction; RCA = right coronary artery.

**Central Illustration:**
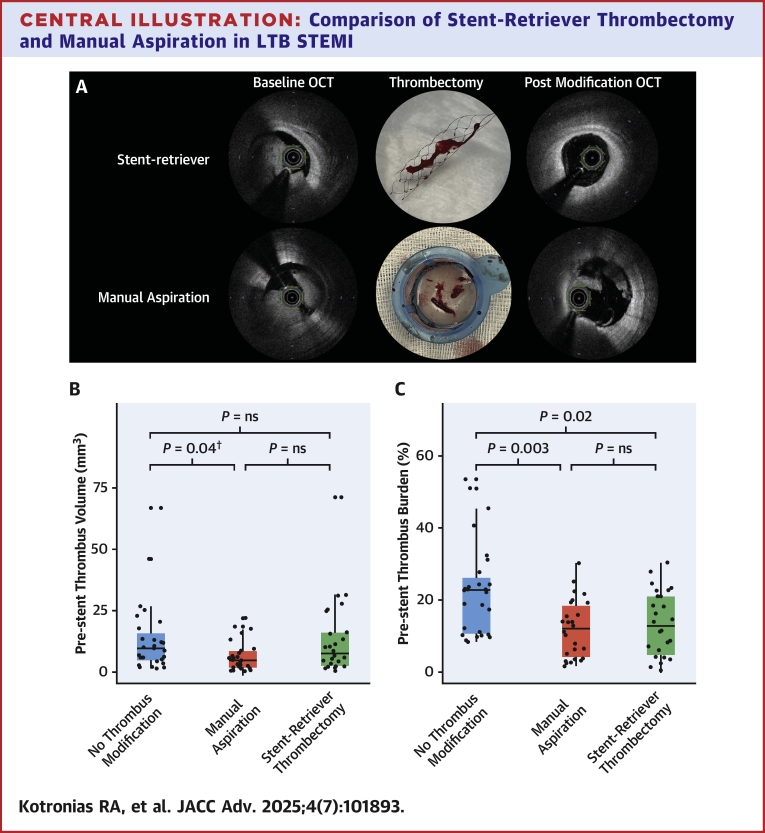
**Comparison of Stent-Retriever Thrombectomy and Manual Aspiration in LTB STEMI** Illustrative cases demonstrating differential efficacy of stent retriever thrombectomy vs manual aspiration via prethrombus and postthrombus modification OCT (A). Effect of thrombus modification strategies on prestent thrombus evaluated by prestent thrombus volume (B) and prestent thrombus burden—thrombus volume/vessel volume (C). ^†^Post hoc analysis with multiplicity adjusted *P* value. LTB = large thrombus burden; other abbreviations as in Figure 1.

### Feasibility and safety of stent-retriever thrombectomy

SRT with the SAVE technique was performed in 26 participants. One participant was not treated as assigned due to significant radial spasm, necessitating the use of a 6Fr guide catheter incompatible with the SAVE technique. There were no challenges encountered with device preparation, advancement, and deployment. The device was deployed once in 25 participants and twice in one case. No device-related clinically relevant arterial injury was detected (dissection/perforation) by angiography or OCT. No periprocedural cerebrovascular accidents occurred.

### Thrombus evaluation by OCT

Diagnostic-quality OCT imaging was achieved in all cases except one in the MA-assisted PCI arm. 7,820 OCT frames were analyzed. The Dice index for frame-level interobserver and intraobserver lumen segmentation similarity was 0.93 (95% CI: 0.92-0.94) (n = 169) and 0.94 (95% CI: 0.93-0.95) (n = 169). The Dice index for frame-level interobserver and intraobserver reliability for thrombus segmentation was 0.74 (95% CI: 0.69-0.0.77) (n = 126) and 0.78 (95% CI: 0.75-0.80) (n = 126).

**Figure 2:**
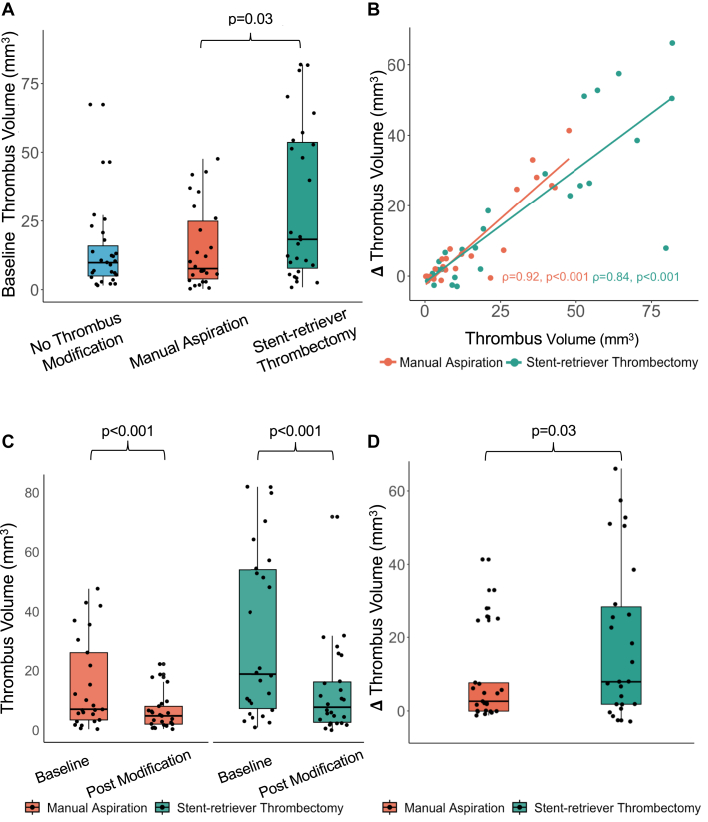
**Evaluating Thrombus Modification Efficacy With OCT** Baseline thrombus distribution across the 3 arms by OCT (A). Correlation between baseline thrombus and the thrombus extracted following stent-retriever thrombectomy and manual aspiration techniques (B). Thrombus burden reduction (C) and comparative extraction efficacy (D) of stent-retriever thrombectomy vs manual aspiration. Abbreviation as in Figure 1.

Baseline thrombus volume varied substantially in the whole study (10.6 mm^3^; IQR: 5.4-26.9 mm^3^; Table 2), although patients with high angiographic thrombus grade had higher baseline thrombus volume (*P* < 0.01). Fourteen participants had a baseline thrombus volume of <3 mm^3^, half of whom had TIMI thrombus grade of 5, with only one case with documented distal embolization prior to OCT imaging. Baseline thrombus volume significantly differed between arms (*P* = 0.04). The SRT arm had significantly higher (*P* = 0.03) baseline thrombus volume than the MA arm, 18.3 mm^3^ (IQR: 6.7-54.4 mm^3^) and 7.6 mm^3^ (IQR: 3.3-27.1 mm^3^), respectively (Table 2, Figure 2A). The lumen volume in the SRT arm was not significantly different to the lumen volume in the MA arm (78.4 mm^3^; IQR: 44.3-145.0 mm^3^ vs 46.0 mm^3^; IQR: 18.6-119.3 mm^3^; *P* = 0.10).

**Table 2:** OCT Evaluation

Baseline	No Thrombus Modification	Manual Aspiration	Stent-Retriever Thrombectomy	*P* Value (Manual Aspiration vs Stent-Retriever)
	(n = 27)	(n = 26)^a^	(n = 27)	
Thrombus volume, (mm^3^)	9.8 (4.5-18.1)	7.6 (3.3-27.1)	18.3 (6.7-54.4)	**0.03**
Flow volume, (mm^3^)	30.6 (19.7-65.0)	37.5 (14.4-93.1)	54.3 (33.7-81.4)	ns
Lumen volume, (mm^3^)	43.9 (24.2-79.2)	46.0 (18.6-119.3)	78.4 (44.3-145)	0.10
Thrombus burden, n (%)	22.8 (10.4-27.7)	22.3 (11.7-31.0)	30.9 (15.1-40.8)	ns
Dissection, n (%)	0 (0%)	1 (3.8%)	1 (3.7%)	ns

Post Modification		(n = 25)^a^	(n = 26)^a^	
Thrombus volume, (mm^3^)	n/a	4.8 (1.8-8.4)	7.7 (2.3-18.6)	ns
ΔThrombus volume, (mm^3^)	n/a	2.6 (0.0-16.1)	8.0 (1.6-31.5)	**0.03**
Flow volume, (mm^3^)	n/a	35.9 (15.1-91.7)	66.5 (41.8-108.6)	ns
Lumen volume, (mm^3^)	n/a	40.9 (19.2-97.6)	70.4 (50.4-136.5)	ns
Thrombus burden (%)	n/a	13.0 (3.8-19.4)	12.8 (4.4-21.5)	ns
Dissection, n (%)	n/a	0 (0%)	0 (0%)	ns

Post Angioplasty	n = 28	(n = 26)^a^	(n = 24)^a^	
Thromboatheroma volume, (mm^3^)	3.4 (1.1-6.3)	1.8 (0.6-4.7)	0.5 (0.0-2.8)	ns
Minimum stent area (mm^2^)	5.9 (4.7-7.1)	6.3 (5.0-7.4)	7.2 (5.4-8.7)	ns
Stend-edge dissection, n (%)	1 (3.6%)	1 (3.8%)	0 (0%)	ns
Malapposition, n (%)	5 (17.8%)	1 (3.8%)	2 (8.0%)	ns
Underexpansion, n (%)	5 (17.8%)	3 (11.5%)	1 (4%)	ns

A *P* value <0.05 denotes statistical significance.

OCT = optical coherence tomography.

a Baseline and postmodification OCT were of nondiagnostic quality in 1 patient in the manual aspiration arm, 1 participant died prior to postmodification OCT in the manual aspiration arm, 1 stent-retriever arm participant was crossed over after baseline thrombus evaluation, while 2 stent-retriever arm participants had nondiagnostic quality OCT post angioplasty.

### Comparative efficacy of devices in thrombus modification

Prestent thrombus volume (Central Illustration B) was not significantly different between SRT (7.7; IQR: 2.3-18.6) and either MA (4.8; IQR: 1.8-8.4; *P* = 0.17) or no thrombus modification (9.8; IQR: 4.5-18.1; *P* = 1.00). However, prestent thrombus burden (Central Illustration C)—measured as thrombus volume normalized to vessel volume—was significantly lower in both the stent-retriever (12.8%; IQR: 4.4%-21.5%; adjusted *P* = 0.016) and MA (13.0%; IQR: 3.8%-19.4%; adjusted *P* = 0.003) thrombectomy arms compared to the no thrombus modification arm (22.8%; IQR: 10.4%-27.7%). Indeed, MA and SRT were efficacious in reducing thrombus volume (Figure 2C, Table 2). A very strong correlation was observed between baseline thrombus volume and Δthrombus volume (rho = 0.87; 95% CI: 0.72-0.96; *P* < 0.001) (Figure 2B). The correlation remained strong when MA and SRT were separately considered (rho = 0.92; 95% CI: 0.82-0.98; *P* < 0.001 and rho = 0.84; 95% CI: 0.63-0.96; *P* < 0.001, respectively, 2B). Notably, SRT extracted a median thrombus volume of 8.0 mm^3^ (IQR: 1.6-31.5 mm^3^), compared to 2.6 mm^3^ (IQR: 0.0-16.1 mm^3^) with MA (Figure 2D). This threefold difference in extraction capacity highlights an important role of SRT (*P* = 0.03) and represents a substantial effect size (Glass’s Δ = 0.77). Finally, residual thrombus volume was not significantly different (*P* = 0.08) between the 2 modification strategies 7.7 mm^3^ (IQR: 2.3-18.6 mm^3^) and 4.8 mm^3^ (IQR: 1.8-8.4 mm^3^) (Table 2).

### Impact of thrombus modification on angioplasty and myocardial perfusion

**Figure 3:**
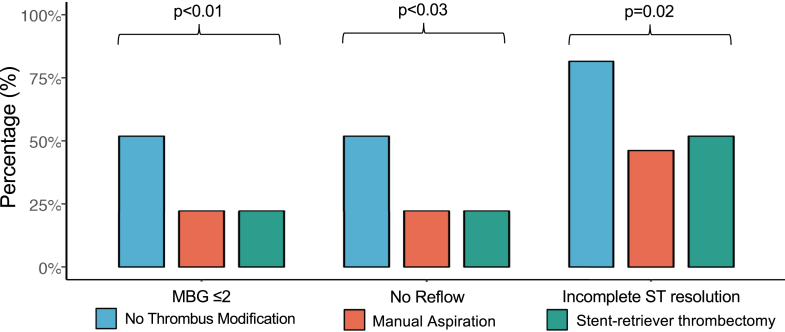
**Impact of Interventional Thrombus Modification on Myocardial Perfusion** MBG = myocardial blush grade.

The incidence of angiographic no-reflow was significantly higher (*P* = 0.03) in the no thrombus modification arm (50.0%) than in either MA-assisted PCI (22.2%) or SRT-assisted PCI arms (18.5%) (Figure 3, Table 3). Bailout GP IIb/IIIa significantly differed between the study arms (Table 3) and was nominally higher in the no thrombus modification and SRT-assisted PCI arms, reflecting the investigational and unblinded nature of the study. However, this does not affect the study’s primary endpoint, as it is assessed before GP IIb/IIIa administration. MBG, final TIMI flow, and IMR_angio_ results are presented in Table 3 and Supplemental Figure 3. The rate of complete ST resolution was significantly less in the no thrombus modification arm (Table 3, Figure 3). Overall, an interventional thrombus modification strategy was associated with improved myocardial perfusion. Finally, there was no significant difference between the arms in distal embolization, malapposition, stent edge dissection, or underexpansion (Table 3), although there was a significant difference in poststent thromboatheroma prolapse favoring SRT when compared to no thrombus modification (*P* < 0.01).

**Table 3:** Periprocedural Outcomes

	No Thrombus Modification (n = 27)	Manual Aspiration (n = 27)	Stent-Retriever Thrombectomy (n = 27)	*P* Value (Across Arms)
Procedural aspects				
Bailout GP IIb/IIIa, n (%)	13 (50.0%)	4 (15.3%)	10 (40%)	**0.02**
Arterial dissection, n (%)	1 (3.7%)	1 (3.9%)	1 (3.7%)	ns
Coronary perforation, n (%)	0 (0%)	0 (0%)	0 (0%)	ns
Distal embolization, n (%)	2 (7.4%)	0 (0%)	2 (7.4%)	ns
CVA/TIA, n (%)	0 (0%)	0 (0%)	0 (0%)	ns
Angiographic perfusion				
Final TIMI flow, n (%)				0.08
0	0 (0.0%)	1 (3.7%)	0 (0.0%)	
1	0 (0.0%)	2 (7.4%)	1 (3.7%)	
2	10 (37.0%)	2 (7.4%)	4 (14.8%)	
3	17 (62.9%)	22 (81.4%)	22 (81.4%)	
Myocardial blush grade, n (%)				**<0.01**
1	5 (18.5%)	6 (22.2%)	2 (7.4%)	
2	9 (33.3%)	0 (0.0%)	4 (14.8%)	
3	13 (48.1%)	21 (77.7%)	21 (77.7%)	
Angiographic no-reflow, n (%)	14 (51.8%)	6 (22.2%)	6 (22.2%)	**<0.03**
Final IMR_angio_, AU^a^	50.0 (35.8-74.0)	41.0 (30.9-47.9)	40.6 (35.7-50.1)	ns
Final IMR_angio_ >40 AU, n (%)^a^	17 (73.9%)	12 (54.5%)	11 (57.9%)	ns
ECG				
Complete ST resolution, n (%)	5 (18.5%)	14 (53.8%)	13 (48.1%)	**0.02**

AU = arbitrary units; CVA = cerebrovascular accident; ECG = electrocardiography; IMR_angio_ = angiography derived index of microvascular resistance; ns = not significant *P* > 0.10; TIA = transient ischemic attack; other abbreviation as in Table 1.

a IMR_angio_ analyzable in 23 no thrombus modification arm participants, 22 manual aspiration arm participants and 19 stent-retriever thrombectomy arm participants.

### Clinical outcomes

Follow-up was completed in 79/81 patients due to 2 withdrawals of consent at follow-up. In-hospital and 6-month clinical outcomes did not differ between arms (Table 4).

**Table 4:** Clinical Outcomes

In-Hospital	No Thrombus Modification	Manual Aspiration	Stent-Retriever Thrombectomy
	(n = 27)	(n = 27)	(n = 27)
Mortality, n (%)	0 (0.0%)	1 (3.7%)	0 (0.0%)
Pulmonary edema, n (%)	1 (3.7%)	2 (7.4%)	0 (0%)
Cardiogenic shock, n (%)	1 (3.7%)	1 (3.7%)	0 (0.0%)
CVA/TIA, n (%)	0 (0%)	0 (0%)	0 (0%)

6 Months	(n = 26)	(n = 26)	(n = 27)
All-cause mortality, n (%)	2 (7.7%)	1 (3.8%)	0 (0.0%)
Cardiac mortality, n (%)	1 (3.8%)	1 (3.8%)	0 (0.0%)
Heart failure, n (%)	1 (3.8%)	1 (3.8%)	0 (0.0%)
Revascularization, n (%)	1 (3.8%)	1 (3.8%)	0 (0.0%)

Abbreviations as in Table 3.

## Discussion

RETRIEVE AMI is the first randomized study to establish the feasibility and safety of SRT with the Solitaire X revascularization device in patients with STEMI and large angiographic thrombus burden. Second, while no difference in prestent thrombus volume was observed with SRT—likely due to baseline thrombus volume imbalance—both stent-retriever and MA thrombectomy significantly reduced thrombus volume, with an efficacy proportional to the baseline thrombus volume as evaluated by OCT. No device-related clinically relevant arterial injury was detected and in-hospital and 6-month MACCE did not differ between arms. A sensitivity analysis adjusting for baseline thrombus volume imbalance by normalizing thrombus volume to vessel volume confirmed that both modification strategies significantly reduced prestent thrombus burden when compared to no modification, strengthening confidence in the efficacy of SRT. Moreover, the head-to-head comparison between stent-retriever and MA thrombectomy demonstrated that SRT extracted substantially larger volume of thrombus than MA thrombectomy. In addition, we observed an important discrepancy between angiographic thrombus burden and absolute thrombus volume as quantified by intravascular imaging. Finally, this is the first randomized trial in STEMI with a LTB to explore the impact of thrombus modification strategy, showing its association with improved myocardial perfusion compared to no thrombus modification.

LTB in STEMI increases procedural complexity (slow flow/no reflow, stent undersizing/malapposition)^18^ and confers an adverse prognosis.^1^^,^^4^ Despite this, treatments targeting LTB such as systemic GP IIb/IIA administration, intracoronary thrombolysis, or MA thrombectomy have shown inconsistent impact on clinical outcomes.^19^ Consequently, their routine use is not recommended by contemporary clinical guidelines,^19^ diminishing the popularity of MA thrombectomy and stifling interest in interventional thrombus modification strategies.^2^ The recent success of novel approaches such as SRT and mechanical sustained aspiration in stroke medicine has revitalized interest in this field. By establishing the feasibility of SRT in STEMI and demonstrating its efficacy in successfully extracting thrombus, we provide the first insights into its potential use in patients with LTB. Importantly, no device-related coronary complications or periprocedural cerebrovascular accidents were observed, corroborating results from a case series exploring the clinical utility of SRT with the NeVA (Vesalio) device.^9^

Our observation that both thrombus modification strategies significantly reduce thrombus burden with increasing efficacy as baseline thrombus burden rises and resulting in a significant difference in prestent thrombus burden compared to no modification, reinforces the role of thrombus modification in LTB cases. Notably, despite enriching for LTB by including patients with a TIMI angiographic thrombus grade of ≥4, a nontrivial proportion of participants had low thrombus burden as quantified by intravascular imaging. This enables us to apply a fresh perspective to the interpretation of the large randomized trials examining thrombus modification in STEMI. Firstly, the patient-level meta-analysis of the TASTE (Thrombus Aspiration in ST-Elevation Myocardial Infarction in Scandinavia), TAPAS (Thrombus Aspiration during Percutaneous Coronary Intervention in Acute Myocardial Infarction Study), and TOTAL (Trial of Routine Aspiration Thrombectomy with PCI versus PCI Alone in Patients with STEMI) trials showed that ∼40% patients randomized to MA thrombectomy had angiographic thrombus burden <4.^4^ The median prestent thrombus burden observed in the TOTAL OCT was 2.36% as opposed to 12.96% observed in our study.^5^ This highlights the drawbacks of the large randomized studies’ patient selection strategy and the role this may have played in the neutral results for MA thrombectomy in STEMI. Additionally, the nominal prognostic benefit of MA thrombectomy observed in a subanalysis of patients with TIMI thrombus grade ≥3 disappeared when the analysis was restricted to grade ≥4 cases.^4^ This unexpected discordance further questions our confidence in angiographic thrombus scoring. In this light, intravascular imaging may help to reliably identify LTB cases for adjunct modification and assess a device's effectiveness for thrombus extraction.

RETRIEVE AMI is the first trial to evaluate device efficacy in thrombus modification using OCT, setting a new benchmark for evaluating device efficacy in thrombus extraction. Both thrombus modification strategies successfully reduced thrombus in proportion to the baseline thrombus. However, SRT demonstrated superior performance, extracting 3 times more thrombus than MA and mitigating chance-related disparities in baseline thrombus. Consequently, the residual thrombus burden was comparably low between the MA and SRT arms and significantly lower than in the no modification arm. Considering the prognostic importance of residual thrombus burden,^7^ SRT appears highly promising for STEMI cases with LTB. While MA thrombectomy remains effective for thrombus extraction, its role may be better suited for cases with moderate thrombus burden.

To date, the value of thrombectomy in patients with LTB has been inferred from post hoc analyses of larger randomized controlled trials showing a nominal prognostic benefit.^4^ In contrast, RETRIEVE AMI is the first randomized study to demonstrate that an interventional thrombus modification strategy in STEMI with a LTB improves myocardial perfusion, establishing a mechanistic link between residual thrombus and impaired perfusion. Notably, effective interventional thrombus modification has recently been linked to reduced myocardial injury and improved clinical outcomes.^6^^,^^7^ While our analyses are hypothesis-generating, the observed no reflow rates are expected due to the enrichment for LTB in our study population and align with existing literature.^20^ Moreover, the high rate of bailout GP IIb/IIIa underscores the challenge of managing LTB and emphasizes the clinical importance of combining pharmacological and interventional approaches to thrombus modification. In this context, our findings highlight the need to revisit and refine thrombus modification strategies for STEMI patients with a LTB. Ultimately, the limitations of the one-size-fits-all approach observed in large-scale randomized trials highlight the importance of leveraging intravascular imaging-based selection strategies for a personalized medicine approach to thrombus modification.

### Study Limitations

RETRIEVE AMI was conducted at a single center and restricted to 3 operators to account for the SRT learning-curve. Secondly, although LTB was selected angiographically using a conservative definition (TIMI thrombus grade ≥4 after wiring), some cases with low thrombus burden, as identified through intravascular imaging, were randomized. This, along with a nominally higher lumen volume in the SRT arm may explain the observed imbalance in baseline thrombus volume between arms. The lack of a detected difference in prestent thrombus volume with SRT likely reflects this imbalance; limiting definitive efficacy comparisons. However, sensitivity analysis confirmed significant prestent thrombus burden reduction with both strategies, supporting the efficacy of SRT. Larger multicenter studies using OCT-based criteria are needed to reduce variability and strengthen comparative assessments. Notably, the discordance between angiographic and intravascular imaging thrombus evaluation may partly relate to instrumentation (predilatation with balloon/OCT advancement) and subsequent distal embolization. However, angiographic distal embolization was observed before OCT in 12 cases, with only one contributing to the discordance.

We acknowledge that we did not directly measure extracted thrombus volume or mass but instead assumed that changes in thrombus volume reflected successful thrombectomy. The absence of worsening microvascular injury, as assessed by IMR_angio_ poststenting, suggests that thrombus removal occurred via stent-retriever/MA thrombectomy rather than distal embolization. This is further supported by the low rate of angiographic distal embolization, making our assumption reasonable, though it may still influence our results. Moreover, we use Δthrombus volume to compare a device’s extraction capacity as a more reliable metric in this context because, unlike OCT-defined thrombus burden (thrombus volume/lumen volume), it is unaffected by thrombus compactness or distribution and remains independent of lumen volume, which can fluctuate due to time-dependent changes in vasomotion, endothelial function, coronary flow, and pressure. Finally, the analyses of clinically relevant myocardial perfusion metrics are underpowered and may be confounded and affected by attrition bias. While they provide valuable insights for planning future research, they should be regarded as primarily hypothesis-generating.

## Conclusions

The RETRIEVE AMI study is the first randomized trial to demonstrate the feasibility and safety of SRT with the Solitaire X device in STEMI patients with LTB. Prestent thrombus volume was not significantly different between SRT and either MA or no thrombus modification, but both modification strategies effectively reduced thrombus, with efficacy proportional to baseline thrombus volume. No device-related clinically relevant arterial injury was detected and in-hospital and 6-month MACCE did not differ between arms. SRT extracted significantly larger thrombus volumes than MA, offsetting chance-related variability. The study highlights discrepancies between angiographic thrombus burden and intravascular imaging with OCT, emphasizing OCT’s potential in identifying high-burden cases, evaluating device performance, and setting a new benchmark for evaluating device efficacy in thrombus extraction. RETRIEVE AMI demonstrates that tailored thrombus modification strategies may improve myocardial perfusion and can address challenges like no-reflow and stent undersizing/malapposition. The findings support future trials exploring the integration of OCT imaging and interventional approaches for personalized management of LTB in STEMI.

Perspectives **COMPETENCY IN MEDICAL KNOWLEDGE**

RETRIEVE AMI is the first randomized trial to establish the safety and feasibility of SRT in STEMI patients with large thrombus burden. Despite not finding differences in prestent thrombus volume with SRT, the study demonstrates the capacity of SRT in extracting larger volumes of thrombus compared to manual aspiration thrombectomy, as assessed by OCT. Finally, thrombus modification is associated with improved myocardial perfusion.

**COMPETENCY IN PRACTICE BASED LEARNING**

TIMI thrombus grade often overestimates thrombus burden when compared to evaluations performed using optical coherence tomography (OCT).

**TRANSLATIONAL OUTLOOK**

Future research should focus on refining thrombus modification strategies for STEMI, integrating intravascular imaging to improve patient selection.

## Funding support and author disclosures

The work was supported by an institutional research grant from 10.13039/100004374Medtronic and 10.13039/501100008645Terumo. Dr Kotronias is supported by the British Heart Foundation grants FS/CRTF/23/24460, the Onassis Foundation and the NIHR Oxford Biomedical Research Centre Imaging and Cardiovascular Themes. Dr Kotronias has received honoraria from Abbott not related to this work. Dr Walsh has received honoraria from Abbott not related to this work. Dr Garcia-Garcia has received institutional grants from Medtronic, Biotronik, Abbott, Neovasc, Corflow, Alucentbio, Philips, and Chiesi not related to this work; consulting fees and honoraria from Boston Scientific, Medis, Abbott, and ACIST not related to this work; support by 10.13039/100008497Boston Scientific, 10.13039/100000046Abbott, and ACIST for attending meetings/travel; and holds a patent (US20210106239A1). Dr Channon is co-founder of Caristo Diagnostics, University of Oxford spin out company. Dr Langrish has received honoraria from Boston Scientific, Abbott, Shockwave Medical not related to this work and support by 10.13039/100008497Boston Scientific, Shockwave Medical for attending meetings/travel. Dr De Maria has received institutional research grants from Opsens; consulting fees from Corflow, Miracor Medical SA; and honoraria from Abbott and Shockwave not related to this work; and holds a patent (US20210106239A1). All other authors have reported that they have no relationships relevant to the contents of this paper to disclose.
