# Supplementary material for: Stent-Retriever Thrombectomy in STEMI With Large Thrombus Burden: The RETRIEVE AMI Randomized Trial
Source: JACC Adv. 2025 Jun 23;4(7):101893. doi: 10.1016/j.jacadv.2025.101893 (PMC12226358; doi:10.1016/j.jacadv.2025.101893)
Supplement: Supplemental Material [file mmc1.pdf]

Stent-retriever Thrombectomy in STEMI with Large Thrombus Burden: The RETRIEVE AMI Randomised Trial

Running Title: Stent-Retriever Thrombectomy in STEMI

Rafail A. Kotronias[a,b,*] MBChB MSc; Jason L. Walsh[a,b,*] MBChB; Stefano Andreaggi[a] MD; Leonardo Portolan[a,b] MD; Alessandro Maino[a,b] MD; Federico Marin[a] MD; Jason Chai[a] MBBCh; Ikboljon Sobirov[b]; Muhammad Sheikh[a] MD; Thomas J Cahill[a] MBBS DPhil; Andrew J. Lucking[a] MBChB PhD; Max Costello[a] BSc; Eva Fraile Moreno[a] MScNurs; Vrinda Haridas[a] BNurs; Anisha Shaji[a] BNurs; Hector M. Garcia-Garcia[c] MD PhD; Keith M. Channon[a,b] MBChB MD; Adrian P. Banning[a,b] MBChB MD; Jeremy P. Langrish[a] MBBCh PhD; Giovanni Luigi De Maria[a,b] MD PhD on behalf of the OxAMI and RETRIEVE AMI Investigators

[a]Oxford University Hospitals NHS Foundation Trust, John Radcliffe Hospital, Oxford, United Kingdom

[b]Acute Multidisciplinary Imaging & Interventional Centre (AMIIC), Division of Cardiovascular Medicine, Radcliffe Department of Medicine, University of Oxford, United Kingdom

[c]Division of Interventional Cardiology, MedStar Cardiovascular Research Network, MedStar Washington Hospital Center, Washington, DC

Corresponding Author Address

Oxford University Hospitals NHS Foundation Trust
John Radcliffe Hospital
Headley Way, Headington
Oxford
OX3 9DU
Email: Giovanniluigi.Demaria@ouh.nhs.uk

Supplement Methods

OCT Analysis

OCT thrombus segmentation was performed according to the methods described in the TOTAL OCT substudy. A frame-by-frame analysis was performed to contour the lumen and thrombus area of pre-PCI runs. Thrombus was defined as an intraluminal mass that was either free floating into the lumen or attached to the vessel wall. The luminal border was traced manually. In frames where the luminal border was not visible the lumen contouring was inferred from the nearest proximal or distal frame with visible lumen contour. Pre- and post-thrombus modification runs of participants who had manual aspiration or stent-retriever thrombectomy were matched using anatomical landmarks as references and a common longitudinal region of interest (ROI) was identified.

Post-PCI runs were also analysed for all 3 arms. Stent struts were identified, and apposition and expansion were characterised. A stent was considered optimised if the minimum stent area was ≥80% of the average reference lumen areas or the minimum stent area was $\geq 4.5mm^2$. Malapposition was referred to as the absence of contact of the stent struts with the vessel wall (>300µm), accounting for strut thickness, which varies according to stent platform adopted. Lumen area (LA), stent area (SA) and thromboatheroma prolapsing areas were traced. Thomboatheroma was defined by the presence of tissue prolapse and thrombus, which was either attached to the vessel wall or the stent struts or separated from it.

For volumetric quantification frame by frame area (mm^2) data of the relevant structure (lumen, thrombus, thromboatheroma) were integrated along the length of the ROI (lumen ROI/

thrombus containing ROI). The length was derived by computing the slice thickness (mm) of each run. Thrombus/thromboatheroma prolapse burden were computed by indexing the thrombus/thromboatheroma prolapse volume to the lumen volume of each run.

Angiography derived IMR (IMR_{angio})

IMR_{angio} at resting conditions was computed, as described previously,(14) using the formula:

$$IMRangio = Pa_{resting} \times QFR_{resting} \times Nframes_{resting}$$

QFR was measured off-line using QAngio® XA 3D software (Medis, Leiden, the Netherlands) by applying it to two angiographic views acquire at 15 frames per second and 30 degrees apart.

Clinical outcomes

Device-related target vessel complications

Device-related target vessel complications include angiographic or OCT identified coronary dissection or perforation, defined and graded according to established classification schemes.(16-18) Device relatedness was adjudicated according to the timing of the complication in relation to the device deployment by expert operators.

Major adverse cardiac and cerebrovascular events

Major adverse cardiac and cerebrovascular events were defined as the hierarchical composite endpoint comprising of death, new or worsening heart failure diagnosis, spontaneous myocardial infarction, target vessel or lesion failure, acute stroke or transient ischaemic attack. All outcomes were adjudicated by an independent clinical event committee.

Heart failure

Heart failure events were evaluated using a standardized heart failure event definition consisting of a) clinical presentation of the subject for new or worsening heart failure symptoms b) objective evidence of new or worsening heart failure, and c) heart failure pharmacotherapy or other treatment.

Symptom criteria:

- Reduced exercise tolerance
- Fatigue
- Dyspnoea
- Evidence of end-organ hypoperfusion
- Hypervolaemia

Signs:

1. Peripheral oedema
2. Increasing abdominal distention or ascites
3. Rapid weight gain attributed to fluid retention
4. S3 gallop
5. Pulmonary rales/crackles/crepitations
6. Raised jugular venous pressure and/or hepatojugular reflux
7. Cardiogenic shock: Sustained (> 30 min) episode of systolic blood pressure < 90 mmHg and/or cardiac index < 2.2 L/min/m2 determined to be secondary to cardiac dysfunction and/or the requirement for parenteral inotropic or vasopressor agents or mechanical support to maintain blood pressure and cardiac index above those specified levels.

Investigations criteria:

1. Increased Brain Natriuretic Peptide/NT-pro Brain Natriuretic Peptide
2. Radiological evidence of pulmonary congestion
3. Echocardiographic findings

Treatments

1. Diuretic treatment (inititation/intensification)
2. Inotropic/vasopressor support
3. Mechanical/surgical support

Periprocedural cerebrovascular accidents

We considered any stroke or transient ischaemia attack occuring within 48 hours from the procedure as periprocedural. Stroke was defined as a new or worsened focal or global neurological deficit of presumed vascular origin, either ischaemic or haemorrhagic, occurring after device deployment and persisting for longer than 24 hours. Transient ischaemic attack was defined as a new or worsened focal or global neurological deficit of presumed vascular origin, occurring after device deployment and persisting for less than 24 hours.

Supplemental Figures

Supplemental Figure 1

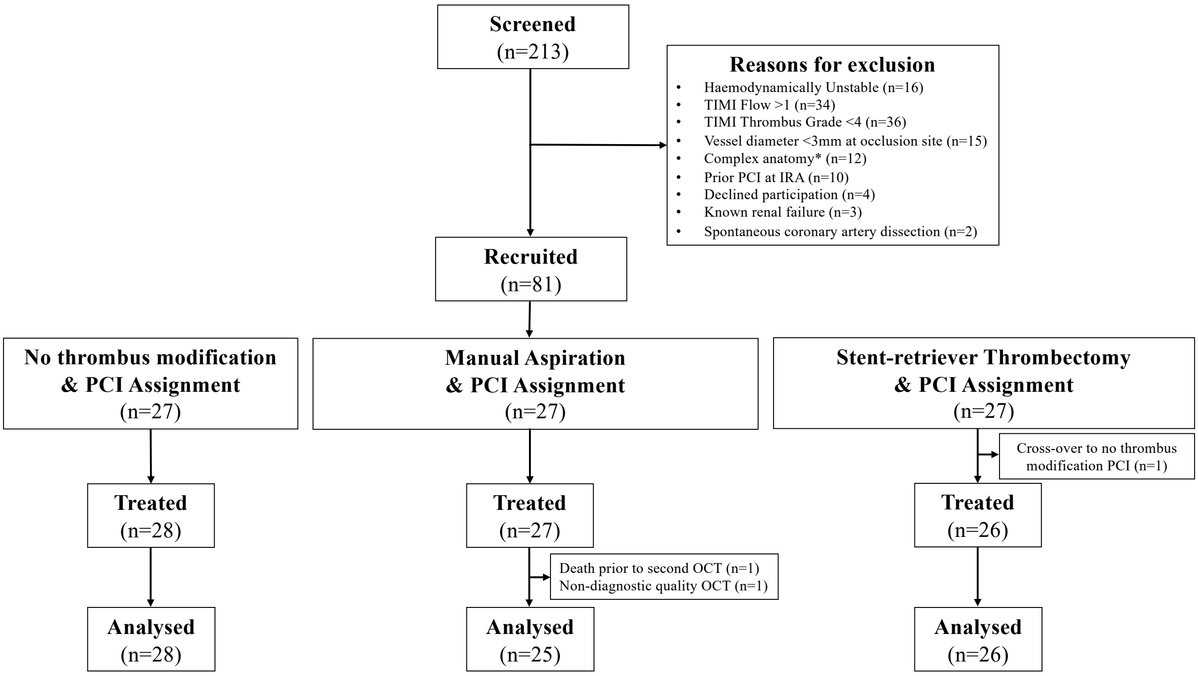

Supplemental Figure 1 – Participant Flow Diagram

IRA: infarct related artery; OCT: optical coherence tomography; PCI: percutaneous coronary intervention; TIMI: Thrombolysis In Myocardial Infarction

Supplemental Figure 2

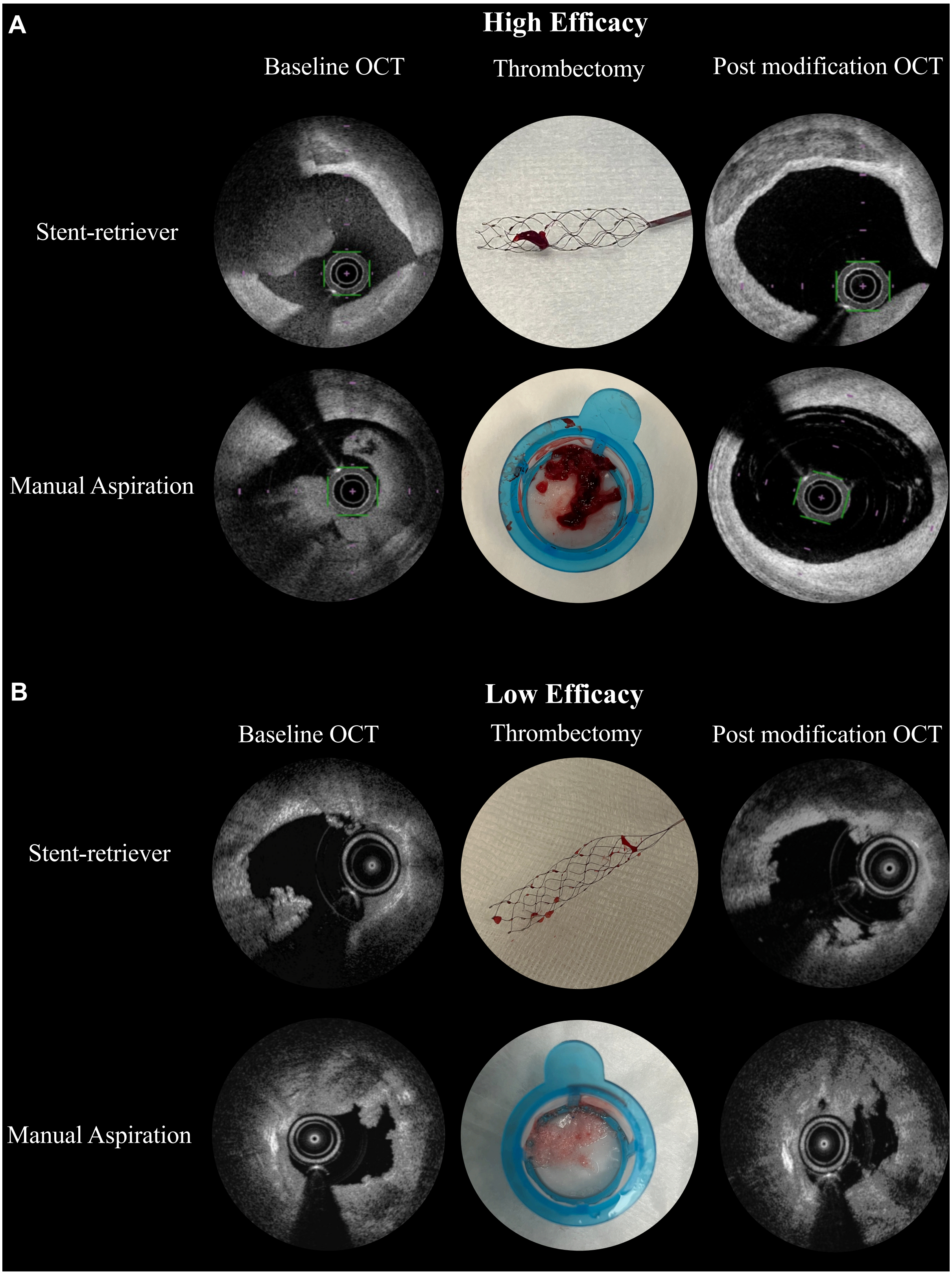

Supplemental Figure 2 – Illustrative cases of thrombus modification efficacy by OCT
Illustrative cases showing high (A) and low (B) efficacy of thrombus modification by stent-retriever thrombectomy and manual aspiration, assessed via pre- and post-modification OCT.

Supplemental Figure 3

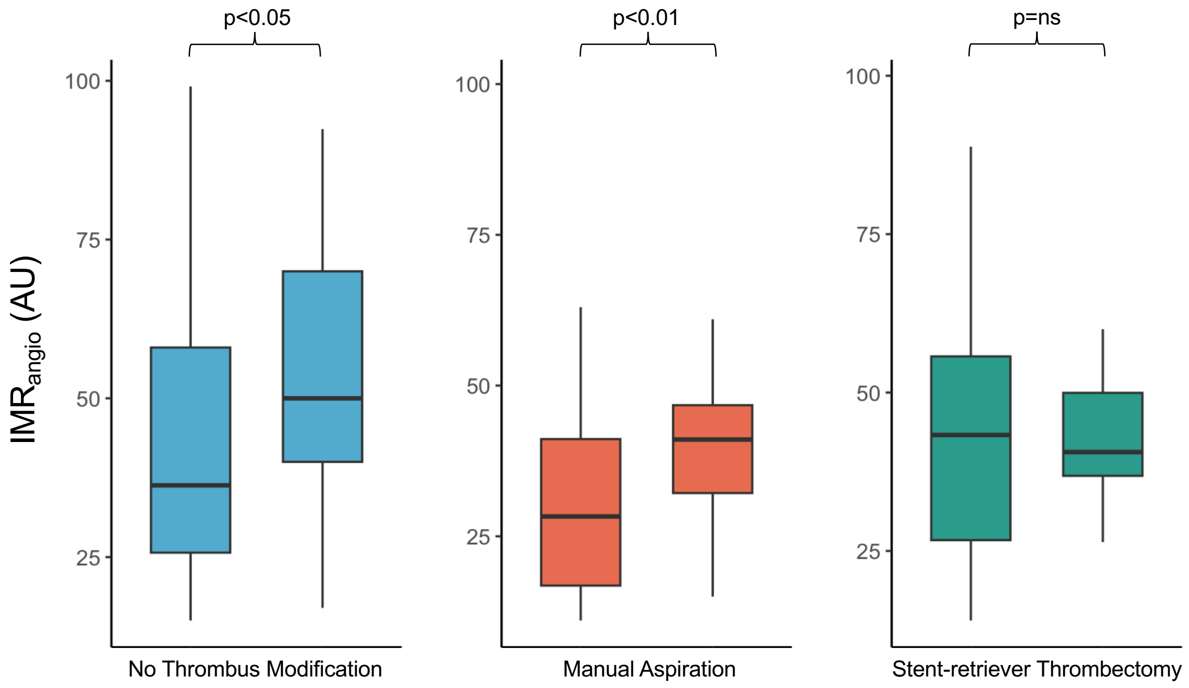

Supplemental Figure 3 – Effect of different strategies of thrombus modification on IMR_{angio}

AU: Arbitrary Units; IMR_{angio}: Angiography derived index of microvascular resistance; ns: not significant.

Section/topic	No	CONSORT 2025 checklist item description	Reported on page no.
**Title and abstract**			
Title and structured abstract	1a	Identification as a randomised trial	1
	1b	Structured summary of the trial design, methods, results, and conclusions	1
**Open science**			
Trial registration	2	Name of trial registry, identifying number (with URL) and date of registration	7
Protocol and statistical analysis plan	3	Where the trial protocol and statistical analysis plan can be accessed	7
Data sharing	4	Where and how the individual de-identified participant data (including data dictionary), statistical code and any other materials can be accessed	7
Funding and conflicts of interest	5a	Sources of funding and other support (eg, supply of drugs), and role of funders in the design, conduct, analysis and reporting of the trial	2
	5b	Financial and other conflicts of interest of the manuscript authors	2
**Introduction**			
Background and rationale	6	Scientific background and rationale	6-7
Objectives	7	Specific objectives related to benefits and harms	10
**Methods**			
Patient and public involvement	8	Details of patient or public involvement in the design, conduct and reporting of the trial	N/A
Trial design	9	Description of trial design including type of trial (eg, parallel group, crossover), allocation ratio, and framework (eg, superiority, equivalence, non-inferiority, exploratory)	7
Changes to trial protocol	10	Important changes to the trial after it commenced including any outcomes or analyses that were not prespecified, with reason	N/A
Trial setting	11	Settings (eg, community, hospital) and locations (eg, countries, sites) where the trial was conducted	7
Eligibility criteria	12a	Eligibility criteria for participants	7
	12b	If applicable, eligibility criteria for sites and for individuals delivering the interventions (eg, surgeons, physiotherapists)	N/A
Intervention and comparator	13	Intervention and comparator with sufficient details to allow replication. If relevant, where additional materials describing the intervention and comparator (eg, intervention manual) can be accessed	8-10
Outcomes	14	Prespecified primary and secondary outcomes, including the specific measurement variable (eg, systolic blood pressure), analysis metric (eg, change from baseline, final value, time to event), method of aggregation (eg, median, proportion), and time point for each outcome	10
Harms	15	How harms were defined and assessed (eg, systematically, non-systematically)	10
Sample size	16a	How sample size was determined, including all assumptions supporting the sample size calculation	12
	16b	Explanation of any interim analyses and stopping guidelines	N/A
Randomisation:			
Sequence generation	17a	Who generated the random allocation sequence and the method used	8
	17b	Type of randomisation and details of any restriction (eg, stratification, blocking and block size)	N/A

			Reported on page no.
Allocation concealment mechanism	18	Mechanism used to implement the random allocation sequence (eg, central computer/telephone; sequentially numbered, opaque, sealed containers), describing any steps to conceal the sequence until interventions were assigned	8
Implementation	19	Whether the personnel who enrolled and those who assigned participants to the interventions had access to the random allocation sequence	8
Blinding	20a	Who was blinded after assignment to interventions (eg, participants, care providers, outcome assessors, data analysts)	11
	20b	If blinded, how blinding was achieved and description of the similarity of interventions	11
Statistical methods	21a	Statistical methods used to compare groups for primary and secondary outcomes, including harms	12-13
	21b	Definition of who is included in each analysis (eg, all randomised participants), and in which group	13
	21c	How missing data were handled in the analysis	13
	21d	Methods for any additional analyses (eg, subgroup and sensitivity analyses), distinguishing prespecified from post hoc	12-13
**Results**			
Participant flow, including flow diagram	22a	For each group, the numbers of participants who were randomly assigned, received intended intervention, and were analysed for the primary outcome	Supplemental Figure 1
	22b	For each group, losses and exclusions after randomisation, together with reasons	Supplemental Figure 1
Recruitment	23a	Dates defining the periods of recruitment and follow-up for outcomes of benefits and harms	7
	23b	If relevant, why the trial ended or was stopped	
Intervention and comparator delivery	24a	Intervention and comparator as they were actually administered (eg, where appropriate, who delivered the intervention/comparator, how participants adhered, whether they were delivered as intended (fidelity))	13-14
	24b	Concomitant care received during the trial for each group	N/A
Baseline data	25	A table showing baseline demographic and clinical characteristics for each group	28
Numbers analysed, outcomes and estimation	26	For each primary and secondary outcome, by group:<br>● the number of participants included in the analysis<br>● the number of participants with available data at the outcome time point<br>● result for each group, and the estimated effect size and its precision (such as 95% confidence interval)<br>● for binary outcomes, presentation of both absolute and relative effect size	29, 30, 31
Harms	27	All harms or unintended events in each group	13
Ancillary analyses	28	Any other analyses performed, including subgroup and sensitivity analyses, distinguishing pre-specified from post hoc	13, 14,15
**Discussion**			
Interpretation	29	Interpretation consistent with results, balancing benefits and harms, and considering other relevant evidence	16-17
Limitations	30	Trial limitations, addressing sources of potential bias, imprecision, generalisability, and, if relevant, multiplicity of analyses	19-20

Citation: Hopewell S, Chan AW, Collins GS, Hróbjartsson A, Moher D, Schulz KF, et al. CONSORT 2025 Statement: updated guideline for reporting randomised trials. BMJ. 2025; 388:e081123. https://dx.doi.org/10.1136/bmj-2024-081123
© 2025 Hopewell et al. This is an Open Access article distributed under the terms of the Creative Commons Attribution License (https://creativecommons.org/licenses/by/4.0/), which permits unrestricted use, distribution, and reproduction in any medium, provided the original work is properly cited.

*We strongly recommend reading this statement in conjunction with the CONSORT 2025 Explanation and Elaboration and/or the CONSORT 2025 Expanded Checklist for important clarifications on all the items. We also recommend reading relevant CONSORT extensions. See www.consort-spirit.org.
